# Three-Dimensional Printing of the Nasal Cavities for Clinical Experiments

**DOI:** 10.1038/s41598-020-57537-2

**Published:** 2020-01-16

**Authors:** Olli Valtonen, Jaakko Ormiskangas, Ilkka Kivekäs, Ville Rantanen, Marc Dean, Dennis Poe, Jorma Järnstedt, Jukka Lekkala, Pentti Saarenrinne, Markus Rautiainen

**Affiliations:** 1 Department of Otorhinolaryngology – Head and Neck Surgery, Tampere University Hospital, Tampere, Finland; 2 Faculty of Medicine and Health Technology, Tampere University, Tampere, Finland; 3 Texas Tech University Health Sciences Center, Lubbock, Texas USA; 4 Ear & Sinus Institute, Fort Worth, Texas USA; 5 Boston Children’s Hospital, Department of Otolaryngology, Boston, Massachusetts USA; 6 Medical Imaging Centre, Department of Radiology, Tampere University Hospital, Tampere, Finland; 7 Faculty of Engineering and Natural Sciences, Tampere University, Tampere, Finland

**Keywords:** 3-D reconstruction, Three-dimensional imaging, Experimental models of disease

## Abstract

3D printing has produced many beneficial applications for surgery. The technique´s applicability in replicating nasal cavity anatomy for clinical use has not been studied. Our aim was to determine whether 3D printing could realistically replicate the nasal cavities and the airflow passing through them from a clinical point of view. We included Cone Beam Computed Tomography (CBCT) scans of five patients with symptoms of chronic nasal congestion. These CBCT scans were used to print plastic 3D prints of the nasal cavities, which were also CBCT scanned and the measurements were compared. The results *in vivo* were higher than the results *in vitro* in maxillary sinus volumes with a ratio of 1.05 ± 0.01 (mean ± SD) and in the nasal cavities with a ratio of 1.20 ± 0.1 (mean ± SD). Linear measurements *in vitro* were very close to those *in vivo*. Rhinomanometric results showed some differences, but rhinomanometric graphs *in vitro* were close to the graphs *in vivo*. 3D printing proved to be a suitable and fast method for replicating nasal cavity structures and for the experimental testing of nasal function. It can be used as a complementary examination tool for rhinomanometry.

## Introduction

Recently, 3D modelling and printing technology have been used in a variety of medical applications, such as surgical planning, design of implants and tissue for individual patients, research and as an educational and training tool^1^. 3D modelling and printing technology have also been used for the planning of implants and operations for craniofacial and skull base pathologies^2^. In addition, the technology has been used in the planning of head and neck tumour surgery^3^. The technology also offers vast possibilities in the field of reconstructive surgery^4^.

To date, however, not all the possibilities offered by 3D printing technology have been utilised in clinical practice. For example, due to the complicated anatomy of the nose, 3D printing technology has not yet been used for modelling of the anatomy of the nasal cavities for clinical purposes or as a tool for the planning of nasal surgical procedures.

Previously, individual models of the anatomy of the nose and nasal cavities were 3D modelled in silicone^5–8^. The production of these models was, however, slow and labourious. These models were used to study airflow in the nasal cavities using liquid and small particles as substances, but the results were not compared with measurements from actual patients. The methods used were particle image velocimetry (PIV) and computational fluid dynamics (CFD), both of which are labourious and time-consuming methods.

In the present study, our main objective was to assess whether 3D printing could be used to realistically replicate the nasal cavities and the airflow passing through them from a clinical viewpoint. We investigate the applicability of plastic 3D prints of the nasal cavities and paranasal sinuses printed from cone beam computed tomography (CBCT) acquired images. A secondary objective was to determine how well the plastic 3D prints corresponded to the nasal function *in vivo*.

## Materials and Methods

CBCT scans of five adult patients with symptoms of chronic nasal congestion were included in this study. In total, ten individual nasal cavities and maxillary sinuses were studied. Exclusion criteria were chronic rhinosinusitis, nasal cavity polyps and tumours. CBCT was used for patient imaging and data acquisition due to its generalised use for this patient group in our hospital. CBCT exposes patients to a relatively low dose of radiation, less than conventional high resolution CT^9^. For CBCT imaging, we used the Scanora™ 3Dx (Soredex, Inc., Tuusula, Finland). The following imaging parameters were used: 0.2 mm CT slice thickness, voxel size 0.2 mm, 90 kVp, 8 mA and 4 s radiation time.

The plastic 3D prints were printed from CBCT scans acquired from the patients. After imaging, the CBCT data were saved in Digital Imaging and Communications in Medicine (DICOM) format. Matlab® software (MathWorks, Inc., Natick, Massachusetts, United States) was then used to process the DICOM images. The CBCT scans were then combined by stacking the 2D image slices, resulting in a 3D model with a voxel size of 0.2 mm in x, y and z directions. Each image slice in the x-y plane was pre-processed by removing noise with a square-shaped average filter and emphasizing the edges in the image using unsharp masking. Then, the tissue types were classified based on voxel light density values. The areas with the density values greater than the values of the areas of the pneumatised volumes were considered solid, and thus the greyscale 3D model was converted to a binary model. The model was corrected by removing small disconnected regions by performing a morphological opening of the 2D image slices along each axis. Finally, the data were saved in Standard Tessellation Language (STL) format for the 3D printing. Before printing, the STL models were processed with Blender software (The Blender Foundation) by using the Remesh operation to fix any possible errors in the models and to make them compatible with the Slic3r software (open source 3D slicing engine) that was used to generate the toolpaths for the printer.

The plastic 3D prints were produced on a Lulzbot® Taz 4 3D printer (Aleph Objects, Inc., Loveland, Colorado, United States) using the Fused Deposition Modeling (FDM) printing technique with a nozzle size of 0.4 mm and a layer thickness of 0.25 mm. In the FDM printing technique, the raw material is deposited through a print head^10^. The extruded string of fused thermoplastic material is immediately bound to the layer below^1^. Polylactic acid (PLA), a commonly used corn-based thermoplastic material for 3D printing, was used as the raw material^10^.

The plastic 3D prints were printed from the level of the nasopharynx to the level of the frontal sinus in 1:1 size. Since it would have been challenging to remove supporting structures afterwards, no supporting structures were generated for the 3D prints. The printing of one plastic 3D print took approximately 48 hours. No additional clean-up of the printed objects was required. However, some printing artefacts were left in the cavities after printing due to the lack of supporting structures for the overhanging parts. When all five 3D prints had been printed, CBCT images were taken of each print for further analysis and to confirm that the prints corresponded with the data of the real patients.

The volumetric measurements of the pneumatised volumes in the patients’ nasal cavities and maxillary sinuses were measured from the patients’s CBCT scans by using pixel light density values from −1000 to −430 Hounsfield units (HU). The same values were also used for measuring pneumatised volumes in CBCT scans in our previous study^11^. The volumetric measurements were made using OnDemand3D™ (version 1.0, CyberMed, Inc., Yuseong-gu, Daejeon, South Korea). For volumetric measurements of the 3D prints, the equivalent pixel light density values for the CBCT scans were defined with the measuring software’s scaling function to be from −1000 to −800 HU due to the printing material used. Similar nasal cavity and maxillary sinus volumetric measurements were evaluated from all CBCT scans. The volumetric software (OnDemand3D™) used in our study often included excess structures in the areas of the volumetric measurements despite the defined values. However, these structures were manually excluded.

**Figure 1:**
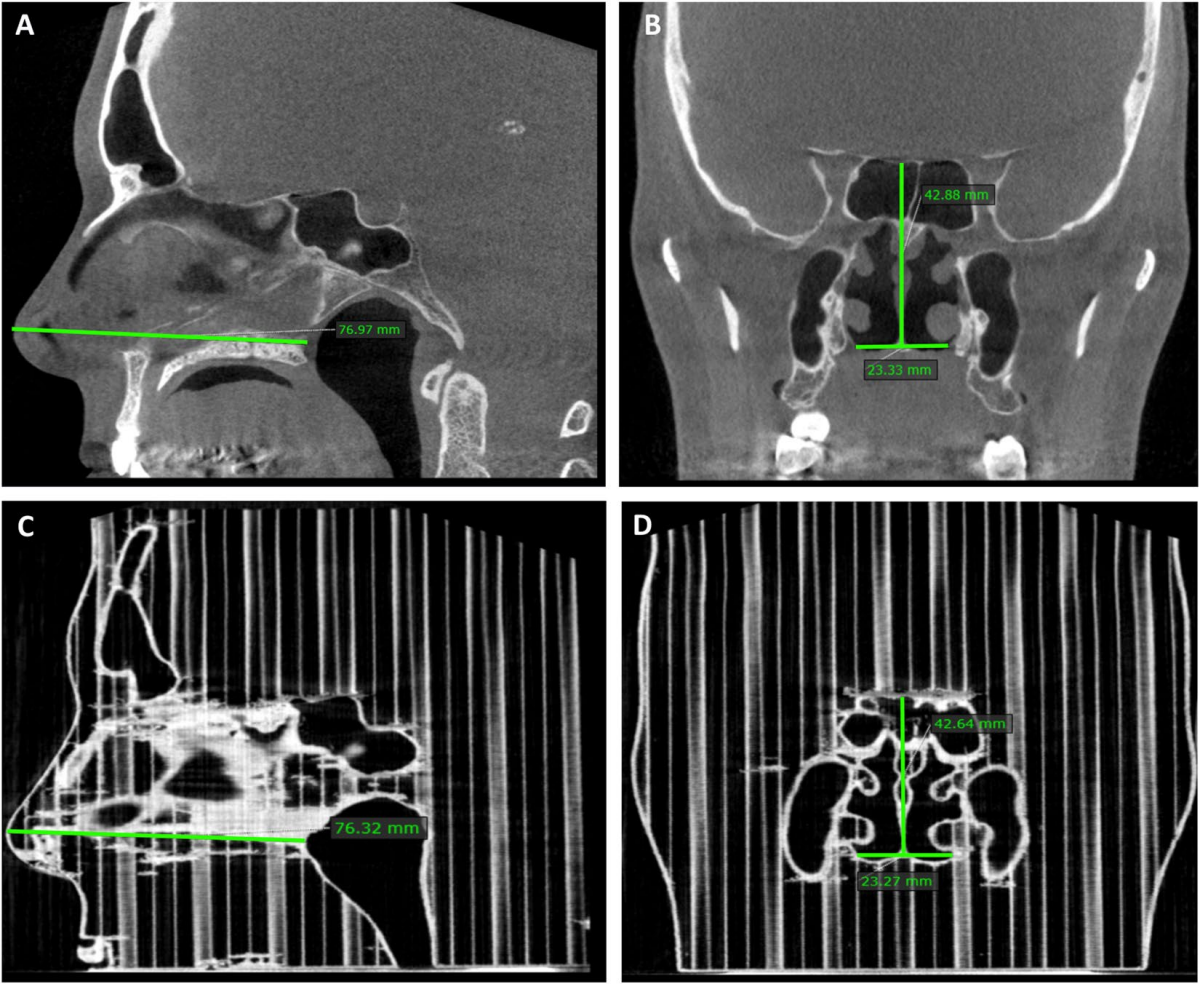
Linear measurements of the nose. Above: Septum length (**A**) and at the first endpoint of the nasal septum both nasal cavity height and width (**B**) in patient CBCT scans. Below: Similar measurements in the corresponding 3D print scans (**C,D**).

Linear measurements in the nasal cavities were made using the same software in all three dimensions in the following way: septum length from the tip of the nose to the closest endpoint of the nasal septum, nasal cavity height at the same endpoint of the nasal septum and the width of the nasal cavity at the same location (Fig. 1). These benchmarks were chosen due to the lower risk of artefacts.

Rhinomanometric resistance measurements of the patients and the 3D prints were also compared. The measurements were done by using anterior rhinomanometry with an NR6 Rhinomanometer (GM Instruments Ltd, Kilwinning, Scotland, United Kingdom) and Nasal Acoustic Rhinomanometer Information System (NARIS) version 3.2 software (GM Instruments Ltd, Kilwinning, Scotland, United Kingdom). The results were measured from the airflow rates by using the Broms method. Rhinomanometry from the 3D prints was done by using the same instrument as used with real patients. In rhinomanometry, the airflow was produced by one of the investigators breathing through a plastic tube connected to the nasopharynx of the 3D prints.

In rhinomanometry, one of the patients and the corresponding 3D print were excluded from this analysis because the patient’s rhinomanometry failed technically. This was noticed after the patient had undergone a nasal operation that resulted in the circumstances in the nose being altered from the original situation, and therefore another rhinomanometry could not be done reliably. As a result, a total of eight individual nasal cavities and maxillary sinuses were studied with rhinomanometry.

Due to the printing induced volume and linear measurement differences observed between the patient and the 3D print measurements, we used a scaling formula to make the rhinomanometric results *in vitro* comparable with the results *in vivo*. Furthermore, we assumed the airflow in the nose being laminar rather than turbulent in the scaling due to the mild flow rates stemmed from the use of Broms method. Thus, the scaling was based on an analogy from laminar tube flow Hagen-Poiseuille equation by taking into account nasal cavity volumes and septum lengths. The scaling for the unilateral rhinomanometric results *in vitro* was calculated by using the following formula derived from Hagen-Poiseuille equation: results *in vitro* multiplied by nasal septum length ratio (*in vivo*/*in vitro*) to the power of three divided by nasal cavity volume ratio (*in vivo*/*in vitro*) squared.

In addition, due to the relatively small number of cases and the deviation of the rhinomanometric results, the use of geometric mean instead of arithmetic mean was found to be more illustrative in presenting the rhinomanometric results. The geometric standard deviation (GSD) was calculated using the following formula: GSD(X) = e^SD(ln X)^, in which e is Euler number, SD is arithmetic standard deviation, ln is natural logarithm and X is measurements.

Institutional review board approval for the study was obtained from Tampere University Hospital, Tampere, Finland. This study was carried out in accordance with the Declaration of Helsinki. Prior to collection of patient data, informed consent was obtained from all participants. All patient data were acquired from the medical records database of Tampere University Hospital.

## Results

We were able to perform reliable measurements both with the patient and 3D print CBCT scans (Table 1). In the volumes of the maxillary sinuses, the results *in vivo* were higher with a ratio of 1.05 ± 0.01 (mean ± SD) compared with the volumetric results *in vitro* (Table 2). In the nasal cavities, the volumetric measurements were higher *in vivo* with a ratio of 1.20 ± 0.1 (mean ± SD) when compared with the results *in vitro*. In every linear measurement, the results were higher in the measurements *in vivo*: 1.03 ± 0.02 (mean ± SD) in nasal septum length, 1.04 ± 0.03 (mean ± SD) in the height of the nasal cavity and 1.06 ± 0.1 (mean ± SD) in the width of the nasal cavity.

**Table 1:** Measurements from every patient and corresponding 3D prints. Rhinomanometric results from patient 5 and corresponding 3D print were excluded due to a technical fail.

	Septum length (mm)	Septum height (mm)	Nasal cavity width (mm)		Maxillary sinus volume (cm³)	Nasal cavity volume (cm³)	Rhinomanometry, inspiration (Pa/[cm³/s])	Rhinomanometry, expiration (Pa/[cm³/s])
Patient 1	81.0	43.2	17.3	Left Right	18.0 17.6	13.2 14.6	0.312 2.199	0.376 2.590
Patient 2	84.8	48.9	10.6	Left Right	16.3 14.4	15.6 12.6	0.212 0.913	0.133 0.884
Patient 3	90.5	45.6	22.9	Left Right	20.7 19.6	19.9 18.5	0.247 0.848	0.331 0.863
Patient 4	80.0	45.2	19.2	Left Right	12.9 12.4	13.2 10.0	0.377 1.297	0.458 1.326
Patient 5	77.0	42.9	23.3	Left Right	24.3 22.6	21.4 19.8	—	—
3D print 1	79.2	40.8	16.6	Left Right	17.1 16.7	10.4 12.1	0.726 0.498	0.708 0.610
3D print 2	82.6	46.5	10.3	Left Right	15.3 13.5	13.0 8.9	0.064 1.617	0.089 1.718
3D print 3	88.8	45.3	23.2	Left Right	20.1 19.0	18.3 16.4	0.527 2.215	0.733 2.202
3D print 4	75.8	42.1	15.5	Left Right	12.3 11.9	11.3 7.8	1.265 2.078	1.222 2.420
3D print 5	76.3	42.6	23.3	Left Right	23.3 21.6	19.4 16.9	—	—

**Table 2:** Mean volumetric (cm^3^) and linear (mm) results (mean ± SD). Five patients (10 maxillary sinuses and nasal cavities, 5 nasal septums) and the corresponding 3D prints are included. In comparison, the mean ratio of the measurements is calculated by comparing the measurements *in vivo* and *in vitro*. Standard deviation of the comparison is shown as percentage points.

	Maxillary sinuses (cm^3^)	Nasal cavities (cm^3^)	Nasal septum length (mm)	Nasal cavity height (mm)	Nasal cavity width (mm)
*In vivo*	17.9 ± 4.0	15.9 ± 3.8	82.6 ± 5.2	45.1 ± 2.4	18.7 ± 5.2
*In vitro*	17.1 ± 3.9	13.4 ± 4.1	80.5 ± 5.3	43.5 ± 2.3	17.8 ± 5.5
Comparison	1.05 ± 0.01	1.20 ± 0.1	1.03 ± 0.02	1.04 ± 0.03	1.06 ± 0.1

**Figure 2:**
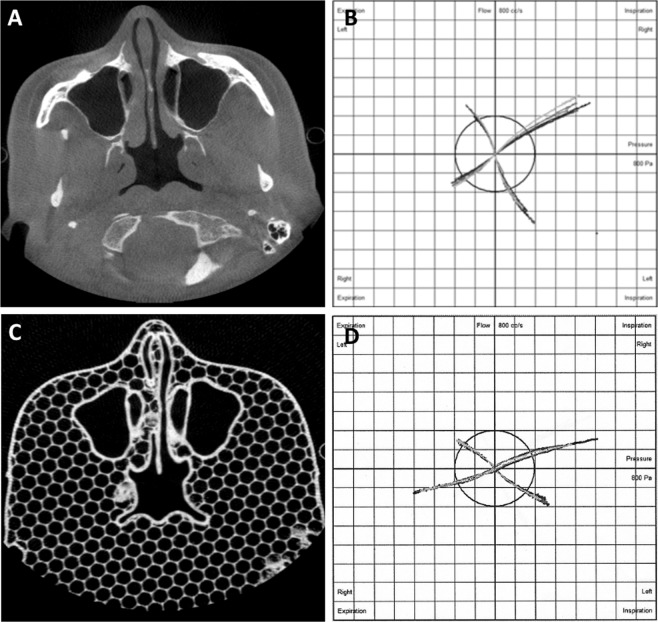
Corresponding results *in vivo* and *in vitro*. Above: Axial CBCT image (**A**) from a patient and rhinomanometry (**B**) from the same patient. Below: Corresponding CBCT image (**C**) and rhinomanometry (**D**) from the 3D print.

In rhinomanometric results, the resistance *in vivo* was less in inspiration with a geometric mean ratio of 0.77 and in expiration with a ratio of 0.71 (Table 3). Standard deviation factors were 2.78 and 2.32, respectively. When the results *in vitro* were scaled based on nasal cavity volume and septum length differences, the similar ratios were 1.03 and 0.95 with standard deviation factors of 2.86 and 2.39, respectively. The total rhinomanometric resistance results were analogous to the unilateral measurement results. The graphs *in vitro* generated by the rhinomanometric software were close to the conditions *in vivo* (Fig. 2). The rhinomanometric graphs are provided more comprehensively in the Supplemetary Information (Suppl. Inf.).

**Table 3:** Geometric mean rhinomanometric resistance measurements (Pa/[cm³/s]). Geometric standard deviation factors are presented in brackets. Inspiratory and expiratory results include eight different nasal cavities from four patients and corresponding 3D prints. The total results from the four patients and 3D prints take both left and right nasal cavities into account. In comparison, the geometric mean ratio of the measurements is calculated by comparing the measurements *in vivo* and *in vitro*. The scaled results *in vitro* and corresponding comparison are also presented.

	Inspiration GM (GSD)	Expiration GM (GSD)	Total inspiration GM (GSD)	Total expiration GM (GSD)
*In vivo*	0.58 (2.34)	0.61 (2.52)	0.23 (1.30)	0.24 (1.65)
*In vitro*	0.76 (3.19)	0.87 (2.89)	0.28 (2.96)	0.33 (2.68)
Comparison	0.77 (2.78)	0.71 (2.32)	0.81 (2.49)	0.71 (1.81)
*In vitro* (scaled)	0.57 (3.15)	0.64 (2.87)	0.22 (3.11)	0.26 (2.84)
Comparison with *in vivo* and scaled results *in vitro*	1.03 (2.86)	0.95 (2.39)	1.04 (2.66)	0.91 (1.97)

GM = Geometric Mean, GSD = Geometric standard deviation factor.

## Discussion

In the present study, we demonstrate the printing of the nasal cavities and maxillary sinuses in 3D prints from CBCT scans acquired from real patients for the first time. Moreover, the quality of the 3D prints were, for the first time, verified by imaging the 3D prints with CBCT and comparing the acquired scans with the patient data. The correspondence between the 3D prints and the original patients in the volumetric and linear measurements was high. Furthermore, we compared the rhinomanometric resistance measurements of the 3D prints to the clinical circumstances. For the first time, in order to better model clinical circumstances, we used a real person’s breathing to provide the airflow of the 3D prints. According to our findings, the accuracy of the 3D prints of the nasal cavities both structurally and functionally are so close to the original circumstances that the 3D prints can be used for the modelling of the nasal cavities *in vitro*.

In the volumetric measurements of the maxillary sinuses and linear measurements, the 3D prints proved to be slightly smaller than the actual patients, although, the differences were small (Table 2). When it comes to measuring the relatively well-bordered anatomical structures using the volumetric method in 3D prints, the results are very close to the conditions *in vivo*. The maxillary sinuses can be accurately isolated for measuring, thus, the volumetric measurements in maxillary sinuses are reliable and can be used in size comparison. In the nasal cavities, artefacts produced in printing the delicate structures of the nasal cavities were the reason for high result in this comparison. Linear measurements also proved to be very close to the original measurements due to the absence of major artefacts in the measuring points of the 3D prints. Moreover, during printing, the size of the 3D prints can be scaled according to the desired size, which gives added versatility to this printing method.

Caution is always required in replicating small and detailed structures as artefacts may be introduced into the 3D print, but despite this the printed result was satisfactory. In our study, the most challenging part of replicating the nasal cavities was to replicate the upper part of the nasal cavity together with the ethmoidal cells. These anatomical structures were vulnerable to any kind of image artefact in the original CBCT scans. In addition, the lack of supporting structures during the printing phase also caused our 3D prints to be exposed to printing artefacts, for example, print surface roughness and narrower structures. Furthermore, we noticed that printing these delicate structures starting from the bottom of the nasal cavity meant that some of the structures were left without any support, and thus, there was the risk of collapse. In most of the models, the upper nasal cavities were narrowed and the inferior nasal conchae had bent under weight. This caused the actual pneumatised area to diminish, which can also be seen in our nasal cavity volume results. These printing technique induced artefacts could most likely be avoided, for example, by beginning printing from above and, if it is practically possible, using soluble supporting structures during the printing process.

Similar observations regarding artefacts with fine anatomical structures were also made in a study where producing 3D prints caused an overall dimensional error of 2.67% for a 3D model of a dry skull^12^. However, the thin bones, small foramina and acute bone projections were not printed as accurately as the rest of the structures.

In the modelling of sinonasal structures, 3D printing would be a reliable tool for research purposes and also an option for PIV experiments and CFD modelling. The 3D printing method is faster, easier and more affordable than PIV and CFD with silicone models, which can take from days to weeks to perform^5–8^. However, PIV experiments do have a future in the exact assessment of airflow in the nasal cavities, which is not possible with the presented method. In our study, the plastic 3D prints were produced in two days. The printing time can be reduced by changing the scale of the 3D prints. The nozzle of the printer and layer thickness can also be changed, thereby affecting the printing time. However, these changes will also have an effect on the surface precision of the 3D prints. In addition, the 3D printing process can be automated, and the procedure can be performed around the clock.

We were able to perform rhinomanometric measurements using the 3D prints. The resistance to airflow proved to be higher *in vitro* compared to the results *in vivo* (Table 3). When the results *in vitro* were scaled based on nasal cavity volumes for a more realistic comparison, the results *in vivo* and *in vitro* were closer to each other. This indicates that rhinomanometric results from various sizes *in vitro* could be scaled with reasonable accuracy to make cases comparable to rhinomanometry *in vivo*.

Rhinomanometric results in our study were likely affected by the properties of the rigid plastic material used as it, in terms of its qualities, was not directly proportional to the actual mucus membrane. In future studies acrylonitrile butadiene styrene (ABS) could be considered an alternative rigid material to PLA. Another option, for example, could be thermoplastic polyurethane (TPU) which is more elastic than PLA. Furthermore, many of the delicate structures in the nasal cavities were troublesome to replicate in printing, with the result of narrowing of some structures that could affect the results. The 3D prints, although printed in 1:1 size, proved to be slightly smaller than the actual patients, which necessitated our use of a scaling formula in our evaluation of the rhinomanometric results.

Our rhinomanometric measurements were conducted with normal breathing. However, temporal information of breathing flow rates was not given by our rhinomanometry device. To be precise, breathing flow rates should be temporally identical between *in vivo* and *in vitro* to be completely certain of a correct comparison. Therefore, the time dependency of the rhinomanometric results should be investigated in future. In a similar way, completely stationary flow resistances should be compared to the present rhinomanometric results. Such studies could reveal important information of how unsteady flow may affect rhinomanometric results. In addition, it has been previously reported that rhinomanometric results *in vivo* could be considerably higher than those obtained by time independent CFD^13,14^. The time dependence of the rhinomanometric results could be an important factor in explaining such differences.

Our 3D modelling method makes all additionally mentioned flow resistance measurements possible. We could expect temporally identical rhinomanometric measurements to reduce the standard deviations in mean ratios between *in vivo* and *in vitro* compared with the present results. Already in the current study, comparison ratios with nasal cavity scaling produced results with reasonable agreement.

## Conclusions

3D printing technology proved to be a suitable and fast method for replicating nasal cavity structures and for the experimental testing of nasal function. The technology enables the detailed study of rhinomanometric measurements, and thus can be used as a complementary examination tool for rhinomanometry for clinical and research purposes. More study to optimise the printing techniques, print materials and modelling processes is warranted to refine this promising model.

## Supplementary information

Supplementary information.

## Data Availability

All data are available on request.
