# Supplementary material for: Three-Dimensional Printing of the Nasal Cavities for Clinical Experiments
Source: Sci Rep. 2020 Jan 16;10:502. doi: 10.1038/s41598-020-57537-2 (PMC6965131; doi:10.1038/s41598-020-57537-2)

**Three-Dimensional Printing of the Nasal Cavities for Clinical Experiments**

Olli Valtonen MD^1,2^*, Jaakko Ormiskangas MSc (Tech.)^2^, Ilkka Kivekäs MD, PhD^1,2^, Ville Rantanen D.Sc. (Tech.)^2^, Marc Dean MD^3,4^, Dennis Poe MD, PhD^5^, Jorma Järnstedt DDS^6^, Jukka Lekkala D.Sc. (Tech.)^2^, Pentti Saarenrinne D.Sc. (Tech.)^7^, Markus Rautiainen MD, PhD^1,2^

¹ Department of Otorhinolaryngology – Head and Neck Surgery, Tampere University Hospital, Tampere, Finland

^2^ Faculty of Medicine and Health Technology, Tampere University, Tampere, Finland

^3^ Texas Tech University Health Sciences Center, Lubbock, Texas, U.S.A.

^4^ Ear & Sinus Institute, Fort Worth, Texas, U.S.A.

^5^ Boston Children’s Hospital, Department of Otolaryngology, Boston, Massachusetts, U.S.A.

^6^ Medical Imaging Centre, Department of Radiology, Tampere University Hospital, Tampere, Finland

^7^ Faculty of Engineering and Natural Sciences, Tampere University, Tampere, Finland

**S1** CBCT scan (A) and rhinomanometric results (B) of the patients (1-5) and the corresponding CBCT scan of the 3D plastic model (C) and rhinomanometric results (D). In the case of patient 5 the rhinomanometric results are excluded because the test had failed technically.

1)


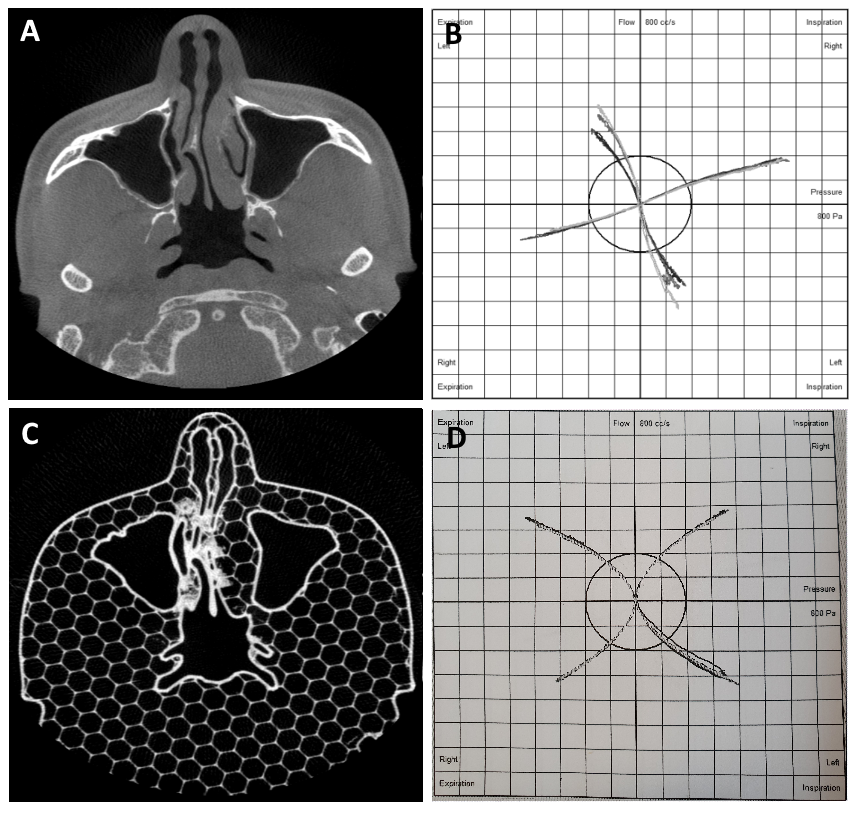


2)


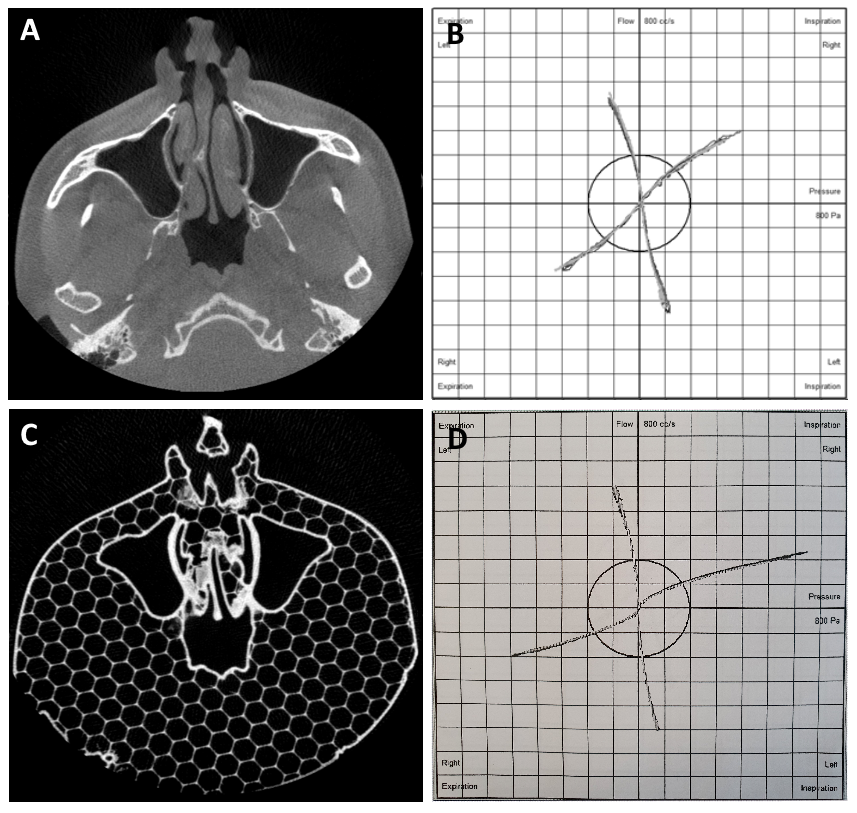


3)


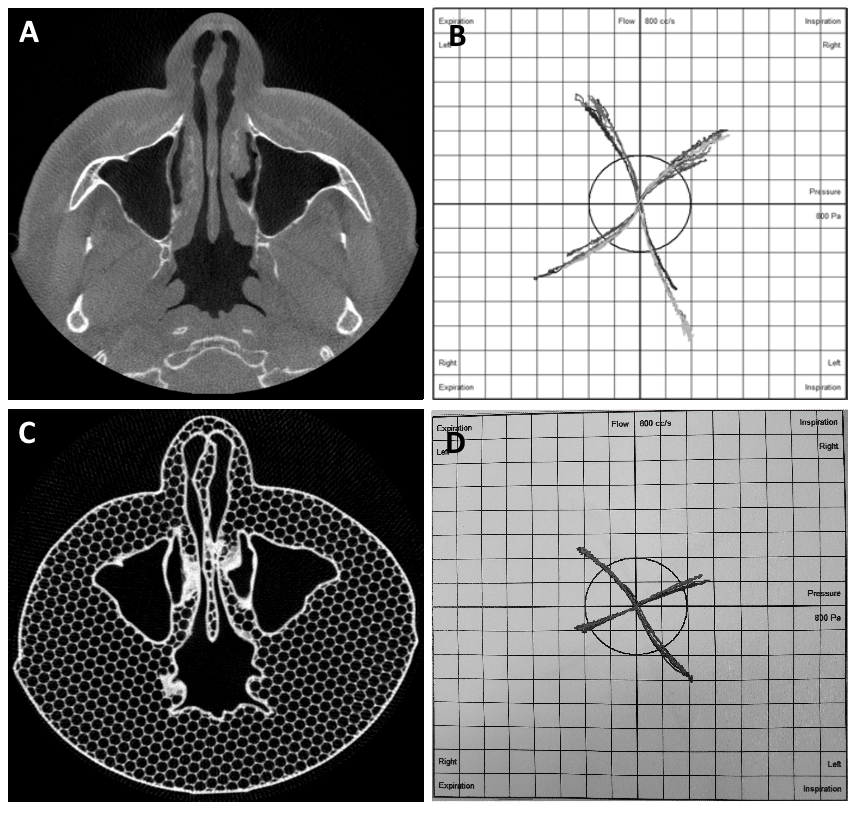


4)


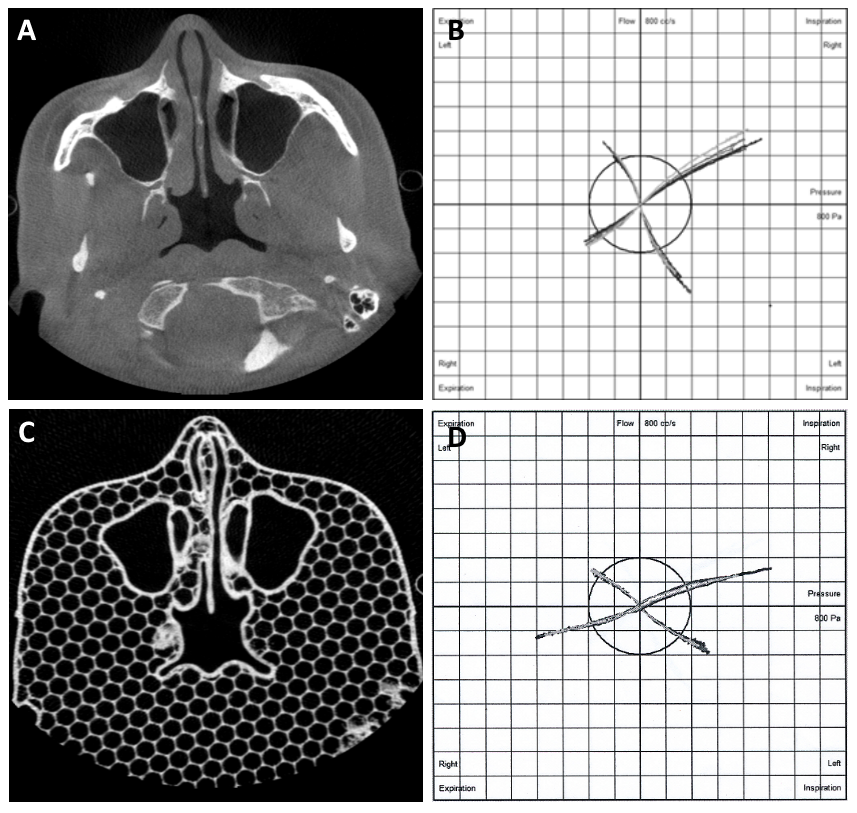


5)


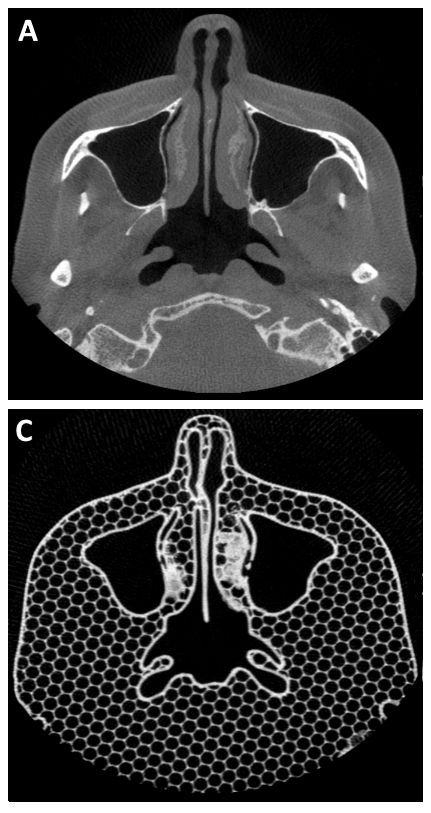

Supplement: Supplementary file 1 — Supplementary information. [file 41598_2020_57537_MOESM1_ESM.docx]
